# *Bellisotoma*, a new genus of Isotomidae from North America (Hexapoda, Collembola)

**DOI:** 10.3897/zookeys.283.3277

**Published:** 2013-04-03

**Authors:** Felipe N. Soto-Adames, Rosanna Giordano, Kenneth Christiansen

**Affiliations:** 1 Illinois Natural History Survey, University of Illinois, Champaign, IL 61820, USA; 2 Department of Biology, Grinnell College, Grinnell, IA 50112, USA

**Keywords:** Lectotype, Acidic sandy soil, Vermont, Quebec, Mississippi

## Abstract

A new genus of Isotomidae, *Bellisotoma* **gen. n.**, is described. The new genus is a member of the *Proisotoma* genus complex and is characterized by a combination of having a bidentate mucro with wide dorsal lamellae that join clearly before the end of mucronal axis without forming a tooth and one strong ventral rib with basal notch that articulates with dens; having abundant chaetotaxy on both faces of dens; and abundant tergal sensilla. Bellisotoma **gen. n.** shows a furcula adapted to a neustonic mode of life, and may be a Isotopenola-like derivative adapted to neustonic habitats. *Subisotoma joycei* Soto-Adames & Giordano, 2011 and *Ballistura ewingi* James, 1933 are transferred to the new genus.

## Introduction

In the process of reviewing new North American species described since the last edition of the Collembola of North America (Christiansen and Bellinger 1998), it became evident that the recently described *Subisotoma joycei* Soto-Adames & Giordano displays a combination of characters that excludes it from any and all currently accepted genera of Isotomidae. Retention of *Subisotoma joycei* in *Subisotoma* Stach would further expand the morphological diversity of species assigned to an already morphological heterogeneous genus. In this contribution, we describe the new genus, add some comments to the original description of *Subisotoma joycei* and transfer *Ballistura ewingi* James to the new genus.

## Results

### Bellisotoma

Soto-Adames, Giordano & Christiansen gen. n.

urn:lsid:zoobank.org:act:AFA4345E-D38F-4D33-8FC4-881181109D23

http://species-id.net/wiki/Bellisotoma

#### Type species.

*Subisotoma joycei* Soto-Adames & Giordano, 2011.

#### Etymology.

The new genus is dedicated to Ross and Joyce Bell, in celebration of their contributions to the study of the entomological fauna of Vermont.

#### Description.

**Figure 1–10.**
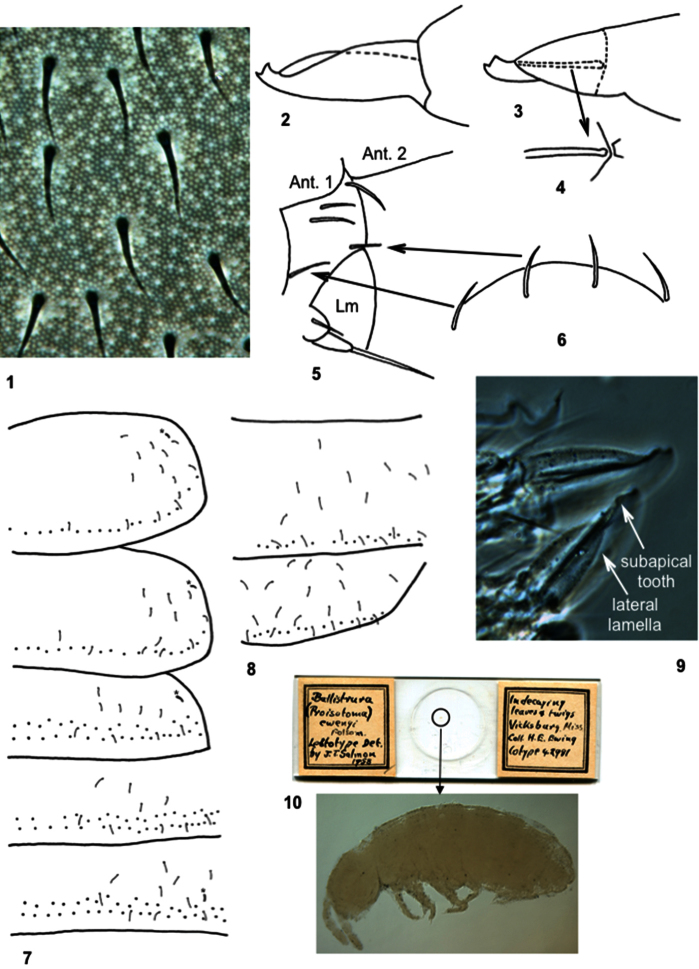
*Bellisotoma joycei*. **1** Ornamentation of dorsal cuticle of head **2** Lateral view of mucro, holotype **3** Oblique view of mucro, specimen from Quebec, hatched lines represent ventral rib and mucronal articulation **4** Ventral mucronal rib and articulation **5** Lateral view of fronto-clipeal region (Ant. 1-2 are 1^st^ and 2^nd^ antennal segments) and labrum (Lm) showing relative placement of prelabral setae **6** Dorsal view of prelabral region, only the inner setae are inserted just basal to labral suture, arrows point at corresponding seta on lateral view of head **7** Chaetotaxy of thorax 2- abdomen 3, asterisks identify microsensilla **8** Chaetotaxy of abdomen 4-5 (from Soto-Adames and Giordano 2011). *Bellisotoma ewingi*, lectotype **9** Structure of mucro (phase/contrast), dorsal and lateral views **10** Scan of lectotype slide and detail of habitus (DIC) showing general condition of specimen.

General body shape short and stout, with sudden bend between abdominal segments 4-5 as in *Folsomides*. Cuticle smooth, granular complex formed by single light granules surrounded by 4-7 darker granules (Fig. 1), granular complexes irregular. Basal microsensilla on antennal segments 3-4 not differentiated; second antennal segment with 3 basal microsensilla; first antennal segment with 17-18 setae, 2 basal microsensilla, 3 basal ventral sensilla and 2 other distal sensilla. Prelabral setae 2; outer maxillary lobe with apical seta simple, sublobal plate with four appendages; labial palp with three proximal setae, all papilla present, guard seta e7 absent. Tergal microsensilla formula 10//101; number of tergal sensilla variable, but adults always with more than eight sensilla on each segment; medial abdominal sensilla inserted either on or just anterior to posterior row (Figs 7–8). Ventral thoracic setae absent. Sterna of second abdominal segment without isolated field of setae. All legs with more than 21 setae; legs with 1-3 weakly capitate or acuminate tenent hairs; setae B4 and B5 present, B5 longer than B4; adult males with setae B5 and x modified. Manubrium without ventral setae. Dens smooth, cylindrical, and shorter than manubrium; dorsal setae long and abundant, distributed throughout dens length; ventral setae few and restricted to distal half of dens. Mucro bidentate (Fig. 2), about half as long as dens, fused to dens dorsally, articulated ventrally; with wide dorsal lamellae that join clearly before the end of mucronal axis without forming a tooth (Fig. 3), and a ventral rib with basal notch that articulates with dens (Fig. 4).

#### Remarks.

*Bellisotoma* gen. n. belongs to the *Isotopenola*-*Subisotoma* genera complex, but unlike these genera, the new genus shows a furcula adapted to a neustonic mode of life, as evidenced by the thick polychaetotic dens and lamellate mucro. *Bellisotoma* gen. n. differs from all other genera by the combination of having a mucro with lamellae that join subapically without forming a tooth, by the presence of a large number of dorsal setae on dens, presence of sensillar polychaetosis, smooth cuticle and second abdominal sternum segment without isolated setae field. The new genus is similar *Isotopenola* Potapov, Babenko, Fjellberg and Greenslade and *Subisotoma*, as circumscribed by Potapov et al. (2009), in having reduced number of prelabral setae, simple outer maxillary palp, reduced number of guard setae on labial papilla E, reduced microsensillar chaetotaxy, absence of tibiotarsal seta B4/5 and smooth dens (Table 1). The three main characters given above distinguish the new genus from *Subisotoma*, whereas the new genus additionally differs from *Isotopenola* in the number of guard setae on labial papilla E (6 in *Bellisotoma*, 4-5 in *Isotopenola*). *Bellisotoma joycei* keys out to *Ballistura* in Potapov (2001), but the two genera are clearly distinguished by maxillary palp structure, sensillar and microsensillar formulae, absence of tibiotarsal seta B4/5 and dens sculpturing. Additional differences between the new genus and other genera in the *Proisotoma* complex (Potapov 2001, Fjellberg 2007, Potapov et al. 2006, 2009) are listed in Table 1. One other North American species, *Ballistura ewingi* (see below), belongs in the new genus.

Members of the new genus may be either psammophilous and/or acidophilus. The individuals of *Ballistura joycei* from Vermont were collected on sandy shores of Lake Champlain and the individuals from Quebec on acidic sandy soils (pH 3.75; Therrien et al. 1996) in a sugar maple grove ≈28Km east of the St. Lawrence River. The exact topotypical locality of *Ballistura ewingi* is not clear, but the soils around Vicksburg, Mississippi are also acidic, sometimes sandy.

**Table 1.** Diagnostic characters for selected genera in the *Proisotoma* genera complex in comparison with *Bellisotoma* gen. n. Characters based on Potapov (2001) or Potapov et al. (2006, 2009). Modified from Soto-Adames and Giordano (2011).

**Genus<br/> Character**	***Scutisotoma***	***Proisotoma***	***Folsomides***	***Ballistura***	***Isotopenola***	***Subisotoma***	***Bellisotoma* gen. n.**
Prelabral Seta	4	3	2	2	2	2	2
Outer Maxillary Palp/Sublobular Appendages	bifurcate/4	simple/4	simple<br/> bifurcate/3	bifurcate/3	simple/4	simple/4	simple/4
Number Guard Setae on Labial Papilla E	7	5	7	6	4-5	6	6
Tergal Microsensillar Formula	11//111	10//000<br/> 00//000	10//000 to<br/> 11//111	11//111	10//101<br/> 11//111	10//000, 10//100, 10//001, 10//101	10//101^a^
Position Medial Sensilla Abd. 3	medial row	posterior row	medial row	just anterior to posterior row	subposterior row	posterior row or just anterior to posterior row	posterior row or just anterior to posterior row
Sternal Thoracic Setae	present<br/> absent	present<br/> absent	absent	absent	absent	absent<br/> present	absent
Mesotibiotarsal Setae B4/5	absent	absent	present	present	absent	absent	absent
Ventral Manubrial Setae	1+1<br/> 0	1+1	0	0	0	0	0
Dorsal/Ventral Dental Setae	variable	3-7/4-6	2-6/0-3	variable	3-8/1	<4/1	18-20/4-6
Tergal Sensilla Polychaetosis	absent<br/> present	absent	absent	absent	absent<br/> present	absent	present
Dental Sculpturing	crenulate<br/> tuberculate<br/> granulate	crenulate	smooth	tuberculate	smooth	smooth	smooth
Mucronal Lamellae	absent	absent	absent	present	absent	absent	present

### Bellisotoma joycei

(Soto-Adames & Giordano), 2011 comb. n.

http://species-id.net/wiki/Bellisotoma_joycei

#### Material examined.

Holotype (Illinois Natural History Survey [INHS] Insect Collection accession number 551,608) and 3 paratypes (INHS Insect Collection accession numbers 551, 610; 551,611; 551, 621): USA,Vermont, Grand Isle Co., South Hero, White’s Beach, N44.62189, W73.32273, sand and thick layer of aquatic plant debris, October 2005. 2 paratypes (INHS Insect Collection accession numbers 551,609 and 551,612): Vermont, Grand Isle Co., Grand Isle, Pearl Bay, west of intersection of East Shore North Rd. and Hide Point West Rd., N44.73078 W73.26401, sand with sparse remains of aquatic plant debris, October 2005. CANADA, Quebec, 9115 (1994), St. Jude, sugar maple leaf litter near N45.78333, W72.93334, Berlese MO-SJ-2, identified as *Proisotoma (Ballistura) ewingi* on the label (this slide contains 7 specimens and is deposited in the A.J. Cook Arthropod Research Collection at Michigan State University).

#### Remarks.

The specimens from Quebec were originally identified as *Ballistura ewingi*, but in the key character that separate *Ballistura joycei* from *Ballistura ewingi* (number of distal setae on the collophore) they are identical to *Ballistura joycei*. All specimens from Quebec are small, the largest measuring only 0.78 mm. Two of the seven individuals from Canada are males. In the largest male the genital plate is well developed, but it appears to be closed, and neither males has modified metatibiotarsal setae. These males are either subadults of adults in reproductive quiescence.

In the specimens from Vermont the number and size of eyes varies, and one individual is blind (Soto-Adames and Giordano, 2011). In the individuals from Quebec the number of eyes is constant, except for one specimen in which eye G is missing on one side of the head. Dorsal views of the prelabral region suggest the presence of 4 prelabral setae (Fig. 6), but a lateral view of the head (Fig. 5) clearly shows that the outer setae are displaced posteriorly, away from the labral suture, and are not prelabral in the usual sense. One individual from Quebec has only one prelabral seta. The number of microsensilla is somewhat variable. Most individuals have 10//10100 microsensilla, but one specimen from Vermont has one microsensillum on the metathorax, and the two males from Quebec have 2 microsensilla on the first abdominal segment.

The shape of the tenent hairs is difficult to ascertain in the specimens from Canada. In the two smallest individuals (0.69 mm) all tenent hairs seem acuminate, whereas in the larger specimens there are 111 capitate and 011 acuminate tenent hairs. Most individuals have 3 tenacular teeth, but two have 3+4 and one has 2+3.

### Bellisotoma ewingi

(James), 1933 comb. n.

http://species-id.net/wiki/Bellisotoma_ewingi

#### Material examined.

Mississippi, Vicksburg; in decaying leaves and twigs, October 2, H.E. Ewing, coll.; Lectotype, designated by J.T. Salmon, 1958; original US National Museum of Natural History catalog number 42981; current catalog number 9026.

#### Remarks.

The description provided by Folsom (1937) suggests that *Ballistura ewingi* and *Bellisotoma joycei* are very similar, sharing characters such as cuticle ornamentation, presence of 3 guard sensilla on the third antennal segment, distal tibiotarsal subsegmentation, and general eye, claw and furcula structure. Of the three main characters used to diagnose *Bellisotoma*, *Ballistura ewingi* has the large number of dental setae and the general lamellate structure of the mucro. The only diagnostic character remaining to be scored for *Ballistura ewingi* is the sensillar polychaetosis.

We studied the lectotype of *Ballistura ewingi*, but the specimen is in such poor condition (Fig. 10) that most characters could not be scored. The few characters we were able to observe are: tergal sensilla present, although clearly seen only on Abd. 4; tibiotarsal setae B4 and B5 present; tenent hair apparently 1,2,2, and acuminate; tenaculum with 3 teeth; dens ventrally with 4 setae; mucro with two lamellae as in Fig. 9.

The tenent hairs and tenacular teeth in the lectotype of *Ballistura ewingi* are as in *Ballistura joycei*, and differ from the numbers reported by Folsom (1937) (2,2,2 or 3,3,3 hairs; 2 teeth). This leaves only the number of distal setae on the collophore to distinguish *Ballistura joycei* (11 setae ) from *Ballistura ewingi* (4 setae), and it is possible that James species is a senior synonym of *Ballistura joycei*. However, the condition of the lectotype is such that we prefer to await the study of fresh material from Mississippi before proposing a definite change in nomenclature. In any case, the similarities between *ewingi* and *joycei* listed above, justify the transfer of *ewingi* to the genus *Bellisotoma*.

## Supplementary Material

XML Treatment for Bellisotoma

XML Treatment for Bellisotoma joycei

XML Treatment for Bellisotoma ewingi

## References

[B1] Christiansen K Bellinger P (1998) The Collembola of North America north of the Rio Grande; A taxonomic analysis. 2^nd^ ed. , Grinnell College, Grinnell, Iowa, 1518 pp.

[B2] Folsom JW (1937) Nearctic Collembola or springtails of the family Isotomidae. United States National Museum Bulletin 168: 1-144. doi: 10.5479/si.03629236.168.1

[B3] James HG (1933) Collembola of the Toronto region with notes on the biology of *Isotoma palustris* Mueller. Transactions of the Royal Canadian Institute 29: 77–116 + 4 plates.

[B4] Potapov M (2001) Synopses on Palaearctic Collembola, Volume 3, Isotomidae. Abhandlungen und Berichte des Naturkundemuseums, Görlitz 73: 1-603.

[B5] Potapov M Babenko A Fjellberg A (2006) Taxonomy of the *Proisotoma* complex. Redefinition of genera and description of new species of *Scutisotoma* and *Webercantha* (Collembola, Isotomidae). Zootaxa 1382: 1-74.

[B6] Potapov M Babenko A Fjellberg A Greenslade P (2009) Taxonomy of the *Proisotoma* complex II. A revision of the genus *Subisotoma* and a description of *Isotopenola* gen. nov. (Collembola: Isotomidae). Zootaxa 2314: 1-40.

[B7] Soto-Adames FN Giordano R (2011) New species of springtails in the *Proisotoma* genus complex from Vermont and New York, USA with descriptive notes on *Ballistura alpa* Christiansen & Bellinger 1980 (Hexapoda, Collembola, Isotomidae). Zookeys 147: 19-37.doi: 10.3897/zookeys.147.2093 PMC3286265 22371662

[B8] Therrien F Chagnon M Hébert C (1999) Biodiversity of Collembola in sugar maple (Aceracea) forests. The Canadian Entomologist 131: 613-628. doi: 10.4039/Ent131613-5

